# Author-suggested reviewers rate manuscripts much more favorably: A cross-sectional analysis of the neuroscience section of PLOS ONE

**DOI:** 10.1371/journal.pone.0273994

**Published:** 2022-12-12

**Authors:** Daniel E. Acuna, Misha Teplitskiy, James A. Evans, Konrad Kording

**Affiliations:** 1 Department of Computer Science, University of Colorado at Boulder, Boulder, Colorado, United States of America; 2 School of Information, University of Michigan, Ann Arbor, Michigan, United States of America; 3 Department of Sociology, University of Chicago, Chicago, Illinois, United States of America; 4 Santa Fe Institute, Santa Fe, New Mexico, United States of America; 5 Department of Neuroscience, University of Pennsylvania, Philadelphia, Pennsylvania, United States of America; Tilburg University, NETHERLANDS

## Abstract

Peer review is an important part of science, aimed at providing expert and objective assessment of a manuscript. Because of many factors, including time constraints, unique expertise needs, and deference, many journals ask authors to suggest peer reviewers for their own manuscript. Previous researchers have found differing effects about this practice that might be inconclusive due to sample sizes. In this article, we analyze the association between author-suggested reviewers and review invitation, review scores, acceptance rates, and subjective review quality using a large dataset of close to 8K manuscripts from 46K authors and 21K reviewers from the journal PLOS ONE’s Neuroscience section. We found that all-author-suggested review panels increase the chances of acceptance by 20 percent points vs all-editor-suggested panels while agreeing to review less often. While PLOS ONE has since ended the practice of asking for suggested reviewers, many others still use them and perhaps should consider the results presented here.

## Introduction

Throughout scientific careers, scientists are routinely asked to propose some reviewers and oppose others when submitting articles or grants. Scientists probably try to suggest competent reviewers, but also want them to appreciate their work. Are these choices desiderable for science or only beneficial for authors? Do suggested reviewers produce better reviews or simply more favorable ones? Answers to these questions could give valuable information to journals and funding agencies about using author-suggested reviewers.

Peer review suffers from many of the same ailments that afflict other human evaluation efforts [1]. Biases might enter the process linked to gender, status, perceived novelty, and positive outcomes [2]. Then there is high inconsistency—a recent experiment showed that pairs of review panels agree not much better than random on acceptance decisions [3]. Moreover, the process is susceptible to manipulation [4], and some evidence suggests that peer review neither improves quality [5] nor predicts future impact [6], although others disagree [7]. Suggested reviewers may exacerbate—or alleviate—these problems.

Recent research has shown that reviewer’s assessment of a manuscript is strongly associated with whether the reviewer was suggested by the authors or not. Several studies have found that suggested reviewers give more positive recommendations than editor-suggested reviewers [8–11], even when the entire review process is public [12]. This author-suggested-reviewer bias also occurs in grant applications [13]. While this association should cause concern, little action has been taken, partly because of the weak evidence demonstrating compromises in review quality [12]. The known association between author-suggested reviewers and their scores deserves more scrutiny.

Here we report the association between author-suggested reviewers and the review process of one of the world’s largest scientific journals—PLOS ONE. We obtained a large sample of manuscripts, and the entire review process of them. We analyze how author-suggested reviewers is associated with timing, scores, acceptances, and review quality as subjectively evaluated by the editor. Overall, we found substantial effects that should be considered in the future.

## Methods and materials

### Data

Through an agreement with PLOS ONE guided by confidentiality and IRB granted through Northwestern University (IRB ID: STU00067837-CR0003, the first two authors of the present study obtained a sample of manuscript reviews from the Neuroscience section (https://journals.plos.org/plosone/browse/neuroscience) of PLOS ONE from January 1st, 2011 until December 31st, 2012. Source data remained encrypted in an off-network computer and noise was added to reviewer decisions to enhance privacy. A 3% chance of randomly changing the review decision of a reviewer for a manuscript revision was added (e.g., randomly choose from reject, major revision, minor revision, and accept). Only after removing identifiers and producing summary statistics were the data moved to the final stage of analyses.

#### The peer review process at PLOS ONE in the Neuroscience section (years 2011 and 2012)

We now explain the review process at PLOS ONE at the time the data were produced. Once manuscripts passed some basic quality and format checks, there were assigned an editor that would overlook the process. At the time of the data (years 2011 and 2012), authors provide three suggested reviewers most of the time during initial submission. Once the editor received the manuscript, she could pick from those reviewers or find suitable reviewers through the internal PLOS ONE editorial manager system, and send an initial set of review requests. As these requests were rejected or became unresponsive, the editor would look for other reviewers.

#### Data description

The data consisted of 7,965 manuscripts from 46,455 authors, 73% of them accepted, which went through 23,964 reviews by 21,665 reviewers. 51% of the manuscripts received a final decision based on a first set of reviews, 40% were based on a second set, and only 8% were based on a third one.

The mean number of author-suggested reviewers per manuscript was 2.89. 46% of the suggested reviewers were invited and, of those, 49% accepted the invitation and provided a review. After each review was received by the editor, the editor could rate the review’s quality using a 0 to 100 scale. Not all reviews received a rating (see Table 6.2 in S1 File), and there were minimal instructions about how to complete this review quality question. Also, the editor knew the type of reviewer (suggested by author, by herself, or opposed reviewer) when evaluating the reviewer’s performance. The variables used throughout the analysis are listed in Table 1.

**Table 1:** Sets of variables analyzed in this article. Some identifiers are shared across these sets of variables and noted when appropriate.

**Reviewers**
Reviewer identifier: identifier of the reviewer across manuscripts and revisions h-index: extracted h-index from Scopus API
**Manuscripts**
Document identifier: identifier used throughout the review process of a manuscript including invitation to review, revisions, and final decision Acceptance: whether the manuscript was ultimately accepted (e.g., has a DOI)
**Reviewer invitations**
Reviewer identifier (see above) Document identifier (see above) Agree to review: whether the reviewer accepted to review the paper Type of reviewer: editor-suggested, author-suggested, or author-opposed
**Reviews**
Document identifier (see above) Reviewer identifier (see above) Revision: revision number (first submission, first revision, second revision, and so on) Revision decision: Reject, Major revision, Minor revision, Accept Editor scores of the reviewer’s reviews: a number from 0 (lowest) to 100 (highest) provided by the editor (optionally) about the outcome of the review process Type of reviewer: editor-suggested, author-suggested, or author-opposed

Seen from the manuscript’s perspective (i.e., author’s perspective), of all manuscripts (authors) who opposed reviewers (23.9%), only 1% of the manuscript had such reviewers invited, and only 0.6% of manuscripts contained reviews from opposed reviewers (Table 2). All manuscripts contained suggested reviewers due to a policy of PLOS ONE at the time. In 70.2% of the cases, such reviewers were invited, and ended up providing reviews for 41.4% of the manuscripts. Editor-suggested reviewers were, in contrast, used in 89.8% of the manuscripts. Seen from the suggestions, invitations, and reviews, we can analyze these numbers from yet another perspective (Table 3).

**Table 2:** Breakdown of reviews and manuscripts per type of reviewer. Percentages are calculated as the fraction of total number of review suggestions/review invitations/reviews, and manuscripts where such information appears per type of reviewer. For example, the first cell (i.e., 5.8% / 23.9%) means author-opposed reviewers were suggested in 5.8% of all review suggestions involving 23.9% of all manuscripts. Similarly, the second cell (i.e., 0.2% / 1.0%) means that author-opposed reviewers were invited to 0.2% of all invitations involving 1.0% of all manuscripts.

	Review suggestions / in # Manuscripts	Review invitations / in # Manuscripts	Reviews / in # Manuscripts
**Author-opposed**	3,893 / 1,902 (5.8% / 23.9%)	112 / 78 (0.2% / 1.0%)	72 / 49 (0.3% / 0.6%)
**Editor-suggested** ^a^	—	37,623 / 7,533 (75.6% / 94.6%)	18,156 / 7,155 (75.8% / 89.8%)
**Author-suggested** ^b^	25,039 / 7,963 (37.6% / 100.0%)	12,036 / 5,588 (24.2% / 70.2%)	5,736 / 3,296 (23.9% / 41.4%)

*Total number of manuscripts: 7,965

^a^Strictly speaking, the editor does not "suggest" but rather immediately invites

^b^PLOS ONE used to require authors to suggest reviewers

**Table 3:** Statistics per manuscript: Number of reviewers suggested by the author, invited, and who provided reviews across revisions. IQR = Interquartile Range.

	Avg	Std	Max	Min	IQR	Median
**Reviewers suggested by author**	3.36	1.85	42	1	2	3
**Reviewers invited**	5.15	3.62	81	1	3	4
**Reviewers who reviewed**	2.07	0.65	9	1	0	2

We provide additional summaries of the data in the supplementary material. For example, information about the authors (Section 3 in S1 File), the review decisions across rounds for different kinds of review panels (Sections 1 and 2 in S1 File), the outcome of manuscripts (Section 4 in S1 File), agreement of reviewers (Section 5 in S1 File), and review quality as judged by the editor (Section 6 in S1 File).

### Methods

#### Matching suggested and opposed reviewers with actual reviewers

The source data already match the submission process with the review process by a unique identifier. However, it does not match the suggested reviewers with the actual reviewers. We performed this matching using a disambiguation package in Python called Dedupe (https://dedupe.io/) which is an open source software for disambiguating based on logistic regression (a form of probabilistic record linkage; see [14] for more details). This provided us with unique identifiers for authors, reviewers, and editors based on their names, affiliations, email, email domain, estimated first, middle, and last names. Using a set of 120 manually labeled datapoints, the method achieved a cross-validated 0.998 recall and 0.999 precision (*F*_1_ = 0.998).

#### Enriching information about authors

The dataset was enriched using the Scopus API (https://dev.elsevier.com/sc_apis.html) with the Author Search functionality through a custom Python package (https://github.com/daniel-acuna/scopus_helper). The author’s full name string and the affiliation were used. The Scopus API matches the name with disambiguated profiles in its database. Sometimes a search would return multiple results with unrelated information. We performed a secondary search on these results to compute simple edit distance matching. After obtaining the Scopus ID of the authors, we use another call to the API to obtain the *h*-index. While Scopus does not capture all the scientific output of authors, especially it does not capture conference proceedings and others, we use the *h*-index for relative comparisons rather than absolute ones.

## Results

The dataset (Table 1) allows us to perform several analyses of the relationship between type of reviewer and outcomes of peer review. In particular, we will analyze the following four outcomes. First, we will analyze the probability that the reviewer invitations are accepted as a function of type of reviewer using reviewer invitations. Second, we will analyze the effects of reviewer type on the revision decision using the reviews. For most analyses, we will only analyze up to the 4th round, which corresponds to more than 95% of all reviews. Third, we will analyze the fate of a manuscript as a function of panel composition across review rounds, with all editor-suggested, all author-suggested, and mixed panels, using a merge between review and manuscript information. Finally, we will examine the review quality of reviews as measured by editors using the reviews’ information. For most of quantities, we report means and standard deviations in addition to medians and IQR. When necessary, we also report effect sizes.

### Review timing and review invitations

**Fig 1:**
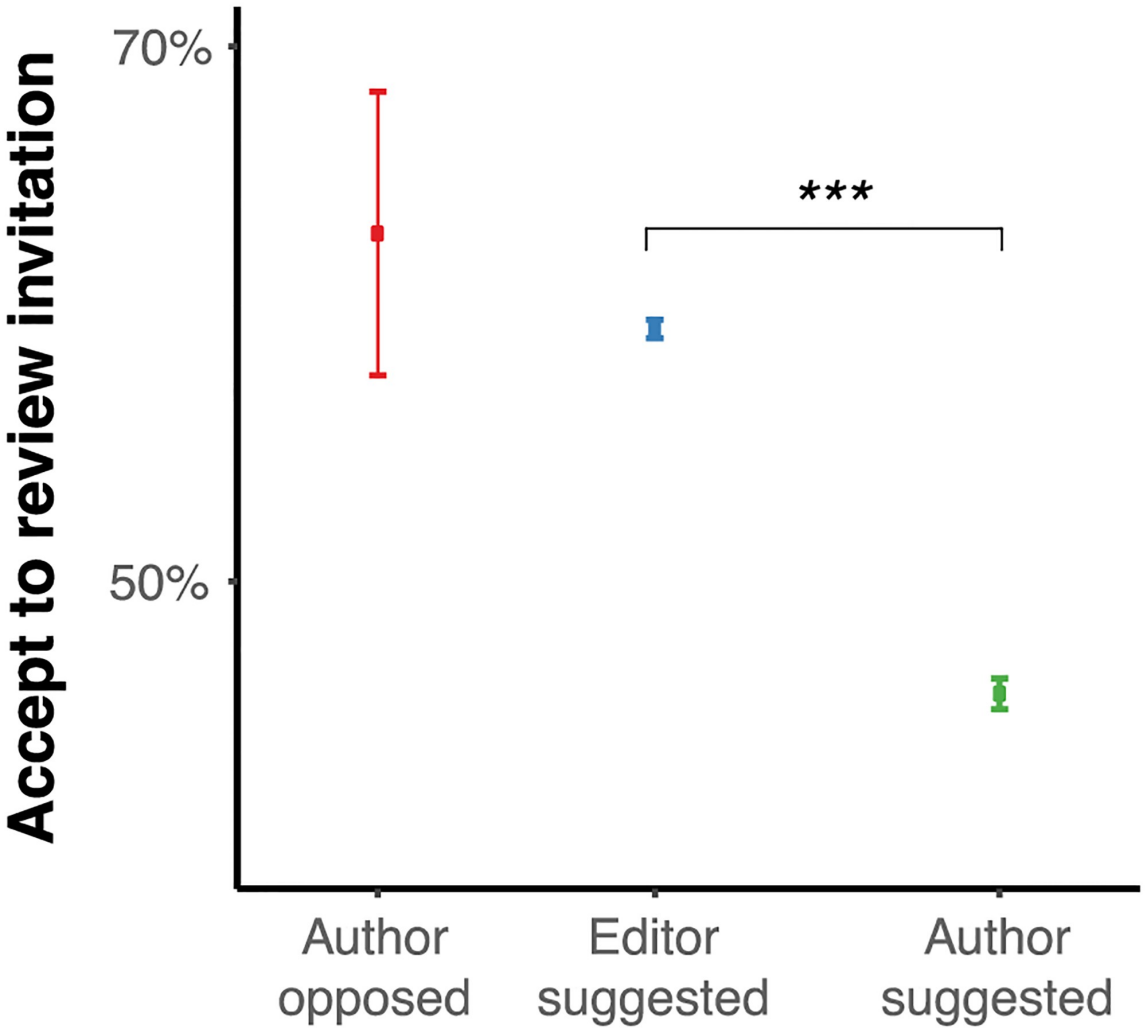
Author-suggested reviewers are less likely to accept a review invitation than editor-suggested reviewers. The point is the *mean* and the error bars are the *standard errors of the mean* (SEM). For comparison across means, the asterisk indicates the statistical significance of a two-sample *t* test where "***", "**", and "*" represent *p* < 0.01, *p* < 0.05 and *p* < 0.1, respectively.

Ideally, the review process should be fast: invitations to review should be accepted with high probability and reviews delivered quickly. We analyze the invitations and timings across all revisions (total sample size) without regard to revision. First, we found that invitation unresponsiveness varied: author-opposed: 8.9%; editor-suggested: 15.8%; author-suggested: 16.5%. Considering only responsive reviewers, we found that there was no significant difference in response time between editor-suggested and author-suggested reviewers (days to respond to invitation: editor-suggested: M = 1.36, SD = 1.08, Mdn = 1.21, N = 7,448; author-suggested: M = 1.35, SD = 1.51, Mdn = 1, N = 5,156; paired diff within manuscript: M = 0.00215, SD = 1.85, Mdn = 0, two-sided paired *t*(4,639) = 0.079, *p* = 0.937) and returned reviews with equivalent speed (days to review: editor-suggested: M = 12.8, SD = 6.75, Mdn = 11.5, N = 7,155; author-suggested: M = 12.6, SD = 7.87, Mdn = 11.5, N = 3,296; paired diff: M = 0.227, SD = 10.7, Mdn = 0, N = 2,488: two-side paired *t*(2,487) = 1.05, *p* = 0.293). Author-suggested reviewers, however, were 13.7% points less likely to accept an invitation (Fig 1, likelihood of accepted invitations within manuscript: editor-suggested: M = 59.2%, SD = 30.7%, Mdn = 57.1%, N = 7,533, author-suggested: M = 45.6%, SD = 42.1%, Mdn = 50%, N = 5,588; paired within manuscript difference: M = 13.7%, SD = 51.8%, Mdn = 18.2%, N = 5,157; two-side paired *t*(5,156) = 19, *p* < 0.001).

### Reviewer recommendations and acceptance rates

**Fig 2:**
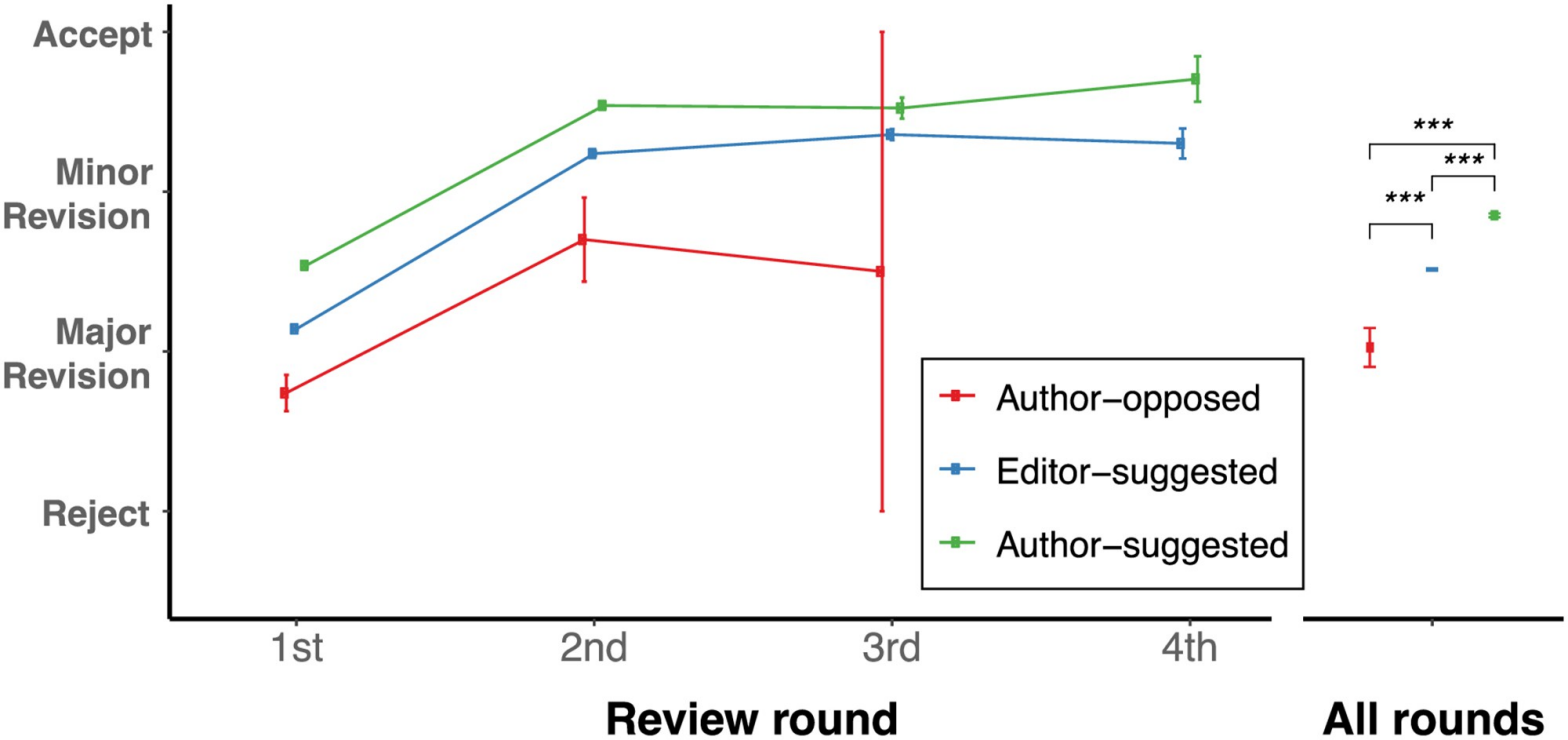
Author-suggested reviewers are more likely to give favorable scores. The point is the *mean* and the error bars are the *standard errors of the mean* (SEM). For comparison across means, the asterisk indicates the statistical significance of a two-sample *t* test where "***", "**", and "*" represent *p* < 0.01, *p* < 0.05 and *p* < 0.1, respectively.

Do author-suggested reviewers provide different evaluations? After codifying revision decisions Rejection, Major Revision, Minor Revision, and Acceptance into an equally separated latent space (e.g., 0, 1, 2, 3, and 4), we estimated statistical differences between scores from reviewers who were author-suggested vs editor-suggested. We ran a regression analysis with score as dependent variable and revision as a categorical control variable to understand the association between type of reviewer across manuscripts and revisions. Using a contrast analysis of the regression results, we found that author-suggested reviewers gave more favorable recommendations than editor-suggested reviewers (contrast = 0.364, *t*(23,954) = 27.4, *p* < 0.001, Cohen’s *d* = 0.415), who in turn were more favorable than opposed reviewers (Fig 2, contrast = 0.453, *t*(23,954) = 4.37, *p* < 0.001, Cohen’s *d* = 0.517). Performing a similar analysis *within* each article, we found that author-suggested reviewers gave more favorable scores than editor-suggested reviewers (two-sided paired t-test: diff = 0.222, *t*(2,487) = 11, *p* < 0.001, Cohen’s d = 0.22), and editor-suggested reviewers gave significantly more favorable scores than opposed reviewers (two-sided paired t-test: diff = 0.802, *t*(41) = 4.36, *p* < 0.001, Cohen’s *d* = 0.673). We additionally performed an ordinal regression to confirm these results, and indeed found similar trends (Table 7.1 in S1 File).

Author-suggested reviewers tended to have a higher *h*-index than editor-suggested reviewers (two-sided paired t-test: diff = 4.04, *t*(1,205) = 7.03, *p* < 0.0001, Cohen’s *d* = 0.202), and therefore it would be desiderable to understand whether this proxy for seniority is causing increases in scores as anecdotally junior scientists tend to be less harsh. A regression analysis on scores revealed that when controlling for seniority, type of reviewer, and revision number, seniority had a small and significant *negative* effect on scores (coeff = -0.0217, *t*(15,716) = -3.06, *p <* 0.001, Cohen’s *d* = 0.024) while most of the positive effect came from whether the reviewer was suggested by the author (coeff = 0.704, *t*(15,716) = 5.88, *p* < 0.001, Cohen’s *d* = 0.784). Taken together, these results suggest that author-suggested reviewers give more favorable decisions and that this favoritism is not a function of *h*-index.

**Fig 3:**
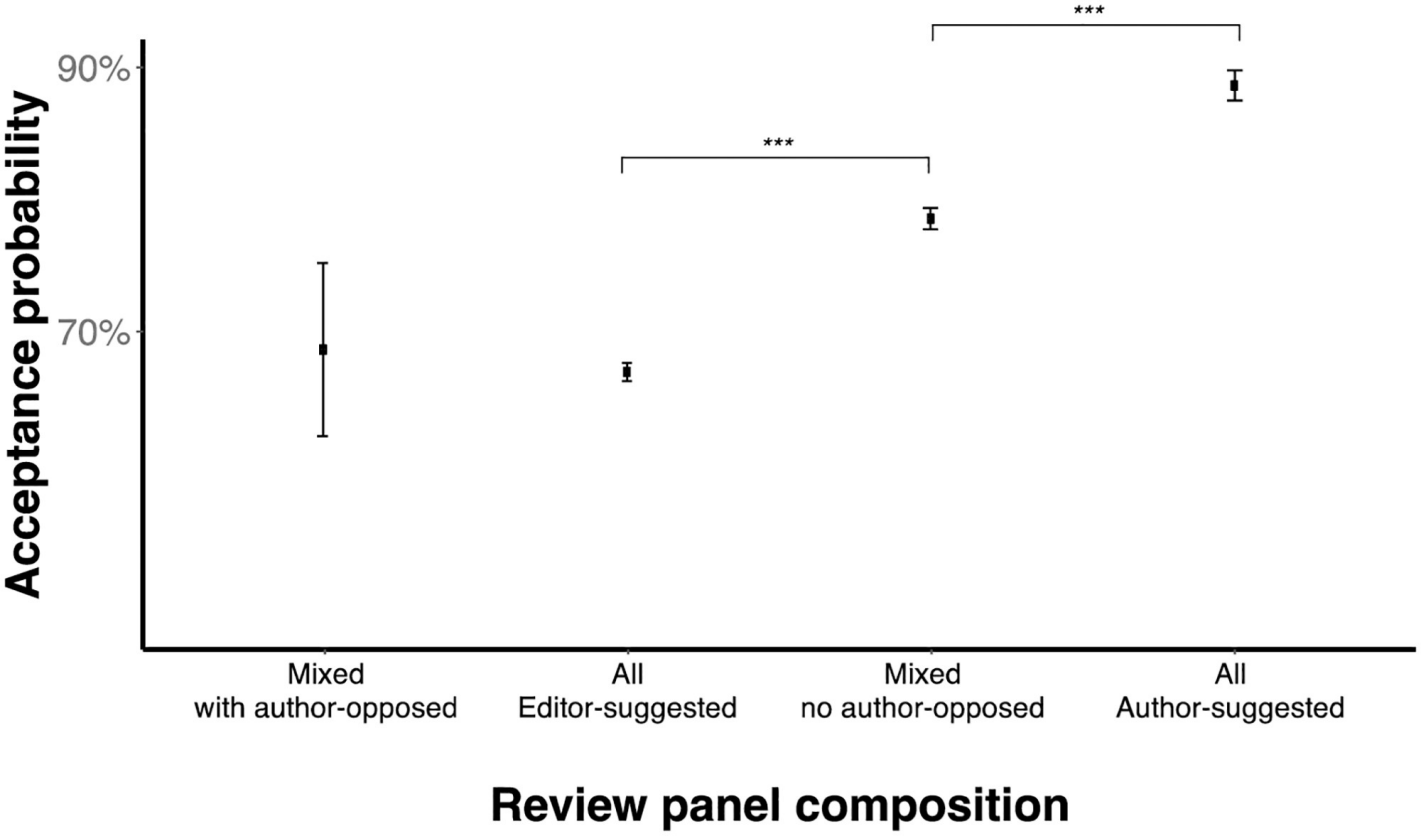
An all-author-suggested panel of reviewers produces a large increase in the acceptance rate of a manuscript. The point is the *mean* and the error bars are the *standard errors of the mean* (SEM). For comparison across means, the asterisk indicates the statistical significance of a two-sample *t* test where "***", "**", and "*" represent *p* < 0.01, *p* < 0.05 and *p* < 0.1, respectively.

Even if individual scores during revisions have potential biases, we might expect editors to take this into account and adjust their final decisions accordingly. To examine this question, we clustered manuscripts into four non-overlapping groups that differ in the composition of the review panel. One category has 4,556 manuscripts where all reviews come from editor-suggested reviewers ("all editor-suggested"). Another category has 762 manuscripts where all reviews come from author-suggested reviewers ("all author-suggested"). Another category has 2,596 manuscripts with mixed review panels with both editor-suggested and author-suggested reviewers but no author-opposed reviewers ("mixed no author-opposed"). Finally, another category has 51 manuscripts with mixed review panels with both editor-suggested and author-suggested reviewers and with at least one author-opposed reviewer ("mixed with author-opposed"). There were no manuscripts with only author-opposed reviewers. Examining the acceptance rates of each of these group can reveal the end effect of different review scores by group. To this end, we performed a logistic regression analysis with dummy variables codifying the type of panel as independent variable and paper outcome as dependent variable. By using planned contrasts, we further compared the difference between panel categories (e.g., all editor-suggested panel vs. all author-suggested panel). Indeed, we found that panel composition is associated with acceptance probability (Fig 3). All editor-suggested panels have, on average, an acceptance rate of 67% (*n* = 4,556), while all author-suggested panels have a significantly higher acceptance rate of 89% (*n* = 762), (planned contrast *t*-test *t*(7,961) = 11.5, *p* < 0.001, two-tailed). Similarly, mixed no author-opposed panels have a significantly higher acceptance rate (79%, *n* = 2,596) vs all editor-suggested panels, (planned contrast *t* -test *t*(7,961) = 10.4, *p* < 0.001, two-tailed). Mixed with author-opposed panels have non-significantly different acceptance rate compare to all editor-suggested panels (69%, *n* = 51) (planned contrast *t*-test *t*(7,961) = -0.257, *p* = 0.797, two-tailed). Therefore, the differences in scores provided by different types of reviewers affects the end acceptance rate of a manuscript.

### Subjective quality evaluation of author-suggested reviews by editor

**Fig 4:**
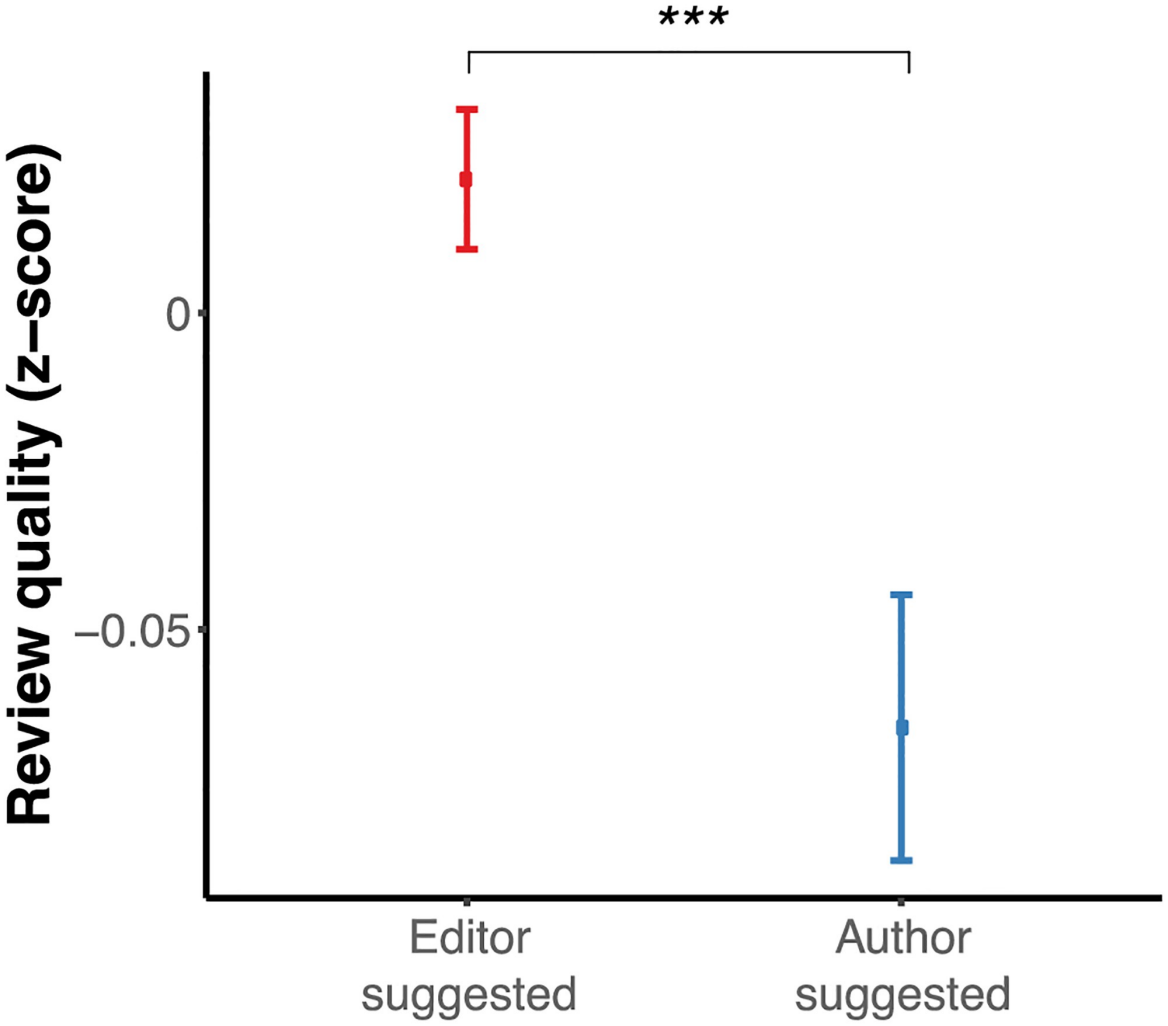
Author-suggested reviewers produce reviewers that are judged as lower quality by editors. The point is the *mean* and the error bars are the *standard errors of the mean* (SEM). For comparison across means, the asterisk indicates the statistical significance of a two-sample *t* test where "***", "**", and "*" represent *p* < 0.01, *p* < 0.05 and *p* < 0.1, respectively.

Despite the differences in decisions and propensity to decline invitations, do author-suggested reviewers provide higher quality reviews that allow editors to reach more meaningful decisions? At PLOS ONE, editors have the option of rating the quality of the reviews (see Materials and Methods, and Table 6.2 in S1 File). Although this rating is not as systematic as previous similar studies (e.g., [11, 15]), it allows to informally investigate this aspect of peer review. We found that, across manuscripts, author-suggested reviewers’ reviews were rated with a significantly lower quality score than those provided by editor-suggested reviewers (Fig 4). A contrast analysis revealed a significant difference but with a small effect size (contrast = 0.08, *t*(10,452) = 3.81, *p* < 0.001, Cohen’s d = 0.08). Within articles, we found the same significant difference, (paired t-test *t*(800) = 3.8, *p* < 0.001, Cohen’s d = 0.134). We should consider that there might be bias in this evaluation because editors knew what kind of reviewer produced the review. This fact likely increased their perception of the quality of the reviews created by their suggested reviewers.

### Dependence on the types of reviewers

There could be hidden causes that are making these differences appear. For example, compared to junior authors, senior authors may know the field better, and have a higher chance of getting author-suggested reviewers to review their manuscripts. To control for this possible confound, we obtained the h-index of the authors, as a proxy for seniority or prestige, and assess the relationship between the maximum *h*-index [16] of the author list and the number of reviewers who are author-suggested. We did find a significant association between of highest *h*-index on acceptance rate (logistic regression analysis: *β* = 5.22 × 10^−3^, 95% *CI* [2.53 × 10^−3^, 7.95 × 10^−3^], *p* < 0.001, *β*_*std*_ = 0.1, 95% *CI* [0.05, 0.16]). However, we found a negative non-significant association between the highest authorship list’s *h*-index and proportion of reviews from author-suggested reviewers (*r*(7385) = -0.017, *p* = 0.12).

## Discussion

In this article, we performed an analysis of the association between author-suggested reviewers and the outcomes of peer review. By analyzing a large dataset of reviews from the PLOS ONE journal, we found that author-suggested reviewers might produce some undesirable outcomes that make the peer review potentially biased. We found that a panel of author-suggested peer reviewers is around 20 percent points more likely to accept a manuscript versus a panel of editor-suggested reviewers while declining more to accept invitations to review. Also, they seem to produce significantly lower quality reviews as subjectively judged by the editors, although we do not have data on how long editors take to search for reviewers not suggested by authors—making it unclear whether the total process becomes slower due to author-suggested reviewers. Overall, these results suggest that journals and funding agencies could benefit from reviewing how the practice of author-suggested reviewers may benefit or hinder them.

One shortcoming of our analysis is that it represents only one journal (PLOS ONE) and one discipline (Neuroscience). Also, PLOS ONE has editorial policies that are substantially different from other journals. Although no longer an active policy, PLOS ONE used to require all authors to suggest reviewers. Importantly, PLOS ONE’s review guidelines are simpler than other journals because reviewers are asked to focus judgment on technical soundness and accurate reporting. Moreover, anything that passes these quality thresholds can be published in PLOS ONE because the virtual journal has no page limit, leading to a relatively high acceptance rate.

Another limitation of our analysis is related to overlapping factors across manuscripts. We are not controlling for reviewers and academic editors being similar across manuscripts. This may introduce some effects in our estimations. For example, reviewers that tend to be author-invited only might review a handful of times but reviewers that are editor-invited might have more experience and be more careful on how they score. Perhaps editors should be aware of these effects and remove them. However, we found that outcome of the review process is still affected by the panel composition. Future studies should investigate the subtler effects of repeated editor-suggested invitees.

Another point of concern is the generalizability of Neuroscience to other fields. However, Neuroscience is relatively large and diverse field, with many different approaches (e.g., experimental vs. theoretical) and points of view [17]. Our results could be applicable to other large disciplines at least. Our findings are also similar to previous findings in other areas [8–11]. While being a specific discipline, Neuroscience thus represents a good sample of scientists.

A possible issue could be that authors that suggest reviewers are fundamentally different from those who do not. For example, reviewers suggested by the authors are more familiar with the topic of the article, and recognize the impact of the work, and therefore are more favorable. Also, reviewers that are senior could receive most of their reviews from author-suggested reviewers. This would explain higher acceptance rates not because of author-suggested reviewers but because of seniority and knowledge of the field. However, we found no correlation between *h*-index and the proportion of reviews produced by author-suggested reviewers.

While the uniqueness of PLOS ONE may hinder the generalizability of our findings, we believe it enhances it in several ways. Any effect of author-suggested reviewers is likely to be more pronounced in journals where more subjective criteria are also considered. PLOS ONE represents a conservative case, as these more subjective criteria (and higher failure rates) would likely amplify the impact of review on the disposition of a considered manuscript. Finally, many fields are represented in PLOS ONE allowing to explore differences in reviewer behavior across disciplines. It should be noted that PLOS ONE changed their review process to disallow author-suggested reviewers in 2014. In the future, we will seek to contrast our findings with those in other journals with more standard practices. For example, other journals ask reviewers to evaluate originality, interest, and relevance, and this can have other distinct effects from the ones found in PLOS ONE [18]. However, we would expect that the lack of these perhaps more subjective goals should make reviewers *less* biases because they only need to consider "hard" evidence when scoring. According to our analysis, however, there seem to be non-trivial effects on the outcomes of review depending on the type of reviewer.

Our results are consistent with other studies in other fields and in other kinds of review contexts. For example, for the journal Atmospheric Science and Physics, a previous study found that author-suggested reviewers do affect the outcomes of peer review [19], finding between 30% and 42% more favorable author-suggested reviews. We found a somewhat smaller favorable increase of 20%. Our analysis involved a significantly larger sample, however. The review quality question was analyzed before by [11, 12] where they found no significant difference between author-suggested and editor-suggested reviews. Our quality measure however was made by the editors whereas in [12] was made by a third party in a more controlled manner. Study [12] found that author-suggested reviewers were more favorable but found that, when author-suggested and editor-suggested disagreed, the editors did not favor the judgement of one or the other type of reviewer. In the context of grants, recent studies have found similar favoritism in grant applications when reviewers are suggested by the applicant or when the institution of the application matches the institution of the reviewer [13]. Thus, the implications of author-suggested reviewers are multi-faceted and deserve further scrutiny.

### Why do editors continue to ask for recommended reviewers?

Editors face a trade off when selecting reviewers. It is easier to choose author-suggested reviewers than find them oneself and author-suggested reviewers might have the right background to appreciate the work under review. We could alleviate the burden on editors by using automated reviewer suggestions, matching reviewers with manuscripts close in topic while controlling for conflicts of interests. Also, we could correct scores when we find measurable evidence of certain biases [20]. The field of Computer Science has been particularly eager to use such systems [21]. We used a similar approach at the Computational and Systems Neuroscience (COSYNE) 2015 conference and found that, topic-wise, automatically chosen reviewers were equally competent to those manually chosen but they felt significantly higher confidence in their scores [22]. Automated systems that aid the review process may be already available, which will make the use of author-suggested reviewers less attractive [23]. For the moment, author-suggested reviewers are an important source of potential reviewer names that perhaps might be used in the future by journals. This is especially true in areas that are more niche and for which editors have a hard time choosing appropriate reviewers. Overall, however, we feel that the practice of allowing authors to propose reviewers should be carefully examined.

## Conclusion

Author-suggested reviewers are in practice in many journals today and our results suggest that can contain some biases. Our findings come from a very large sample compared to previous studies, and they come from a journal that only measures correctness and not significance, novelty, and impact like other journals. Therefore, if anything, PLOS ONE should serve as an interesting control for the cases where peer review should be relatively more objective than other journals. Overall, these results should make us think about the consequence of this practice for science.

## Supporting information

S1 File Contains all the supporting tables and figures. (DOCX)
