# Supplementary material for: Author-suggested reviewers rate manuscripts much more favorably: A cross-sectional analysis of the neuroscience section of PLOS ONE
Source: PLoS One. 2022 Dec 12;17(12):e0273994. doi: 10.1371/journal.pone.0273994 (PMC9744301; doi:10.1371/journal.pone.0273994)
Supplement: S1 File — (DOCX) [file pone.0273994.s001.docx]

Supplementary materials: ­­Should journals allow authors to suggest reviewers?

Daniel E. Acuna^1^, Misha Teplitskiy^2^, James A. Evans^3^, Konrad Kording^4^

^1^School of Information Studies, Syracuse University
 ^2^School of Information, University of Michigan
^3^Department of Sociology, University of Chicago
^4^Department of Neuroscience, University of Pennsylvania

# Distribution of review outcome per type of reviewer and review round

**S1 Figure.** Distribution of review outcomes

**S1 Table.** Distribution of review outcomes

| **Review round** | **Type of reviewer** | **Reject** | **Major Revision** | **Minor Revision** | **Accept** |
| --- | --- | --- | --- | --- | --- |
| **1** | Author-opposed | 46% | 36% | 16% | 2% |
| **1** | Editor-suggested | 24% | 43% | 28% | 5% |
| **1** | Author-suggested | 11% | 34% | 44% | 11% |
| **2** | Author-opposed | 20% | 25% | 20% | 35% |
| **2** | Editor-suggested | 9% | 13% | 24% | 55% |
| **2** | Author-suggested | 4% | 7% | 19% | 69% |
| **3** | Author-opposed | 50% | - | - | 50% |
| **3** | Editor-suggested | 9% | 10% | 18% | 63% |
| **3** | Author-suggested | 5% | 4% | 24% | 67% |
| **4** | Author-opposed | - | - | - | - |
| **4** | Editor-suggested | 8% | 10% | 24% | 58% |
| **4** | Author-suggested | - | 6% | 18% | 76% |

# Distribution of review outcomes per panel compositions

**S2 Table.** Distribution of review outcomes per panel composition

| **Review round** | **Panel composition** | **Reject** | **Major Revision** | **Minor Revision** | **Accept** |
| --- | --- | --- | --- | --- | --- |
| **1** | Mixed with author-opposed | 23% | 40% | 30% | 7% |
| **1** | All editor-suggested | 25% | 43% | 27% | 5% |
| **1** | Mixed no author-opposed | 17% | 39% | 36% | 8% |
| **1** | All author-suggested | 9% | 32% | 47% | 12% |
| **2** | Mixed with author-opposed | 13% | 23% | 13% | 50% |
| **2** | All editor-suggested | 9% | 13% | 24% | 54% |
| **2** | Mixed no author-opposed | 7% | 10% | 21% | 61% |
| **2** | All author-suggested | 3% | 5% | 18% | 74% |
| **3** | Mixed with author-opposed | 14% | 43% | - | 43% |
| **3** | All editor-suggested | 9% | 9% | 19% | 63% |
| **3** | Mixed no author-opposed | 8% | 9% | 19% | 63% |
| **3** | All author-suggested | - | - | 14% | 86% |
| **4** | Mixed with author-opposed | - | - | - | - |
| **4** | All editor-suggested | 11% | 9% | 24% | 55% |
| **4** | Mixed no author-opposed | 2% | 10% | 21% | 67% |
| **4** | All author-suggested | - | - | - | 100% |

# Number of authors per article

**S3 Table.** Authors per article

| Avg authors | Min authors | Max authors | Median authors | IQR authors |
| --- | --- | --- | --- | --- |
| 5.862649 | 1 | 46 | 5 | 5 |

# Rejection after revision decision

**S4 Table.** Fate of articles asked to be revised during first review round

| **Total review rounds** | **Manuscripts** | **Rejected** | **Percent rejected of total^a^** |
| --- | --- | --- | --- |
| 1 | 4045 | 1828 | 23.0% |
| 2 | 3207 | 286 | 3.6% |
| 3 | 613 | 42 | 0.5% |
| 4 | 87 | 8 | 0.1% |
| 5 | 8 | 0 | 0.0% |
| 6 | 4 | 0 | 0.0% |
| 8 | 1 | 0 | 0.0% |
| ^a^ Of total manuscripts: 7965 | | | |

# Inter-rater agreement

**S5 Table.** Inter-rater agreement using Gwet el al. (2008)

| **Review round** | **Two reviewers** | **Three reviewers** |
| --- | --- | --- |
| **1** | 0.211 (*p* < 0.001) | 0.157 (*p* < 0.001) |
| **2** | 0.345 (*p* < 0.001) | 0.220 (*p* < 0.001) |
| **3** | 0.461 (*p* < 0.001) | 0.306 (*p* < 0.01) |

# Review quality per type of reviewer

**S6 Table.** Standardized review scores by editor

| **Type of reviewer** | **Accept** | **Major Revision** | **Minor Revision** | **Reject** |
| --- | --- | --- | --- | --- |
| Author-opposed | 0.10  (SE= 0.6, N=2) | 0.10  (SE=0.2, N=14) | -0.22  (SE=0.44, N=6) | -0.08  (SE=0.4 , N=13) |
| Editor-suggested | -0.12  (SE= 0.03, N=1,253) | 0.14  (SE= 0.01, N= 2,809) | -0.04  (SE=0.02, N=2,195) | 0.01  (SE= 0.02 , N= 1,639) |
| Author-suggested | -0.15  (SE=0.04, N=598) | 0.10  (SE=0.03, N=703) | -0.11 (SE=0.03, N=988) | -0.16  (SE= 0.07 , N= 235) |

**S7 Table.** Percent of total and number of reviews with editor's score

| **Review round** | **Reviews with score** |
| --- | --- |
| **1** | 32.3% (N= 7,744) |
| **2** | 10.0% (N= 2,390) |
| **3** | 1.2% (N= 285) |
| **4** | 0.1% (N= 31) |

# Ordinal regression reviewer decision vs. type of reviewer and review round

We created an ordinal regression on the reviewer decision as a function of the type of reviewer and review round (Table 7.1). Similarly, to the main text, we found that the odds ratio of the editor suggested (using as baseline the author-opposed) 2.58, and the author suggestion is larger still (again compared to author-opposed) at 5.52. As the odds ratio relative different between these two levels is about the same (i.e., 2.6), we think it is reasonable that our main analysis used a linear codification of the decision within a simple linear regression.

**S8 Table.** Ordinal regression outcome

|  | **Decision** | | |
| --- | --- | --- | --- |
|  | *Odds Ratios* | *CI* | *p* |
| Thresholds  Reject \| Major Revision | 0.87 | 0.56 – 1.36 | 0.550 |
| Major Revision \| Minor Revision | 4.75 | 3.04 – 7.41 | **<0.001** |
| Minor Revision \| Accept | 25.07 | 16.04 – 39.19 | **<0.001** |
| Predictors  Type of reviewer [Editor-suggested] | 2.58 | 1.65 – 4.03 | **<0.001** |
| Type of reviewer [Author-suggested] | 5.52 | 3.53 – 8.63 | **<0.001** |
| Review round | 6.78 | 6.43 – 7.15 | **<0.001** |
| Observations | 23,964 | | |
| R^2^ Nagelkerke | 0.272 | | |

# Model checks for regressions

In this section, we do standard checks for the two regression analyses present in the text: regression for analyzing effect of type of reviewer and review round on the decision and regression for analyzing effect of h-index, type of reviewer, and revision number on review outcomes.

## Regression 1

**S2 Figure.** Model diagnostics plot before correction


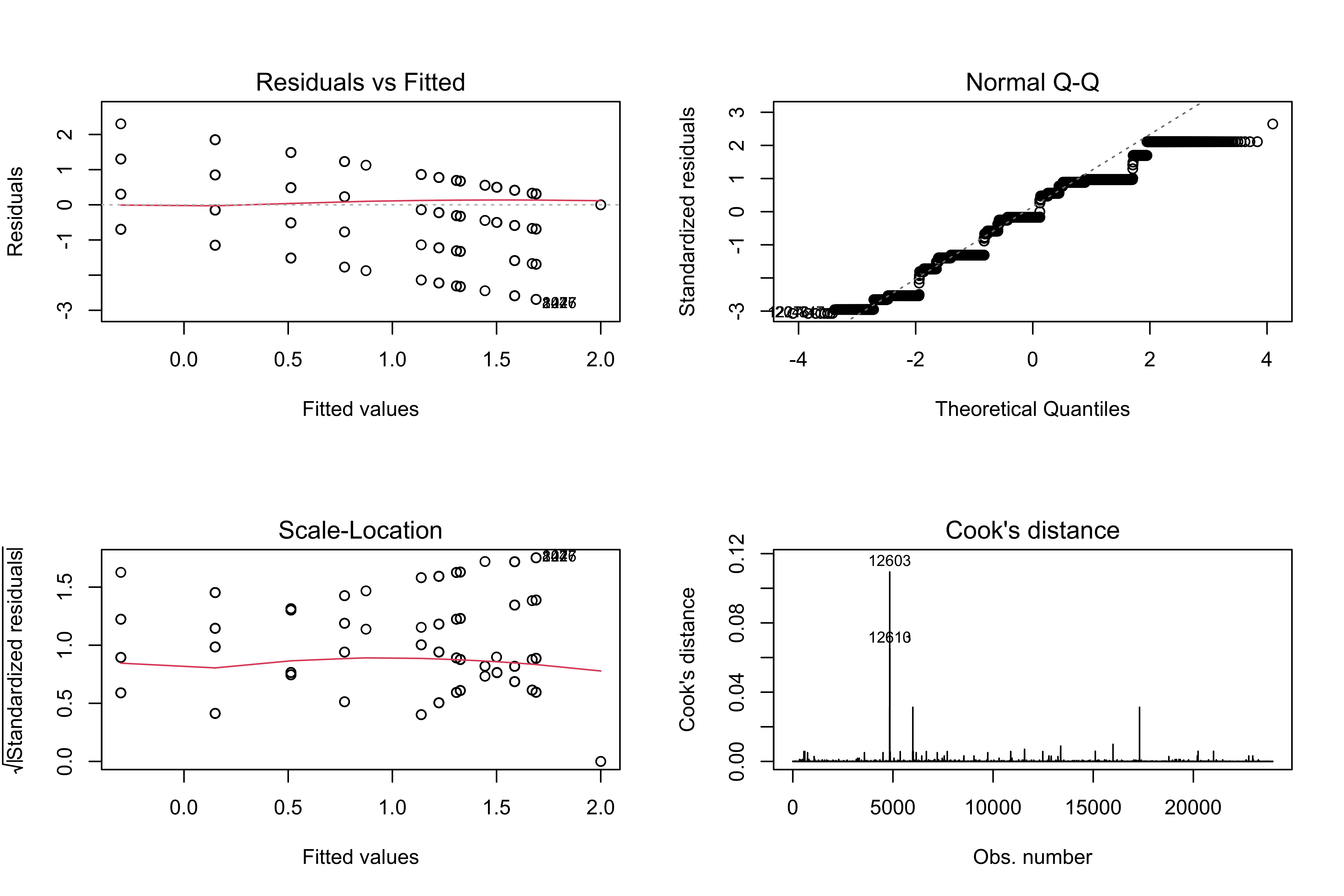


We identify points of high leverage (i.e., potentially influential) to be 2,829. After removing these points, we re-run the regression with the following diagnostics plot:

**S3 Figure.** Model diagnostics plot after correction


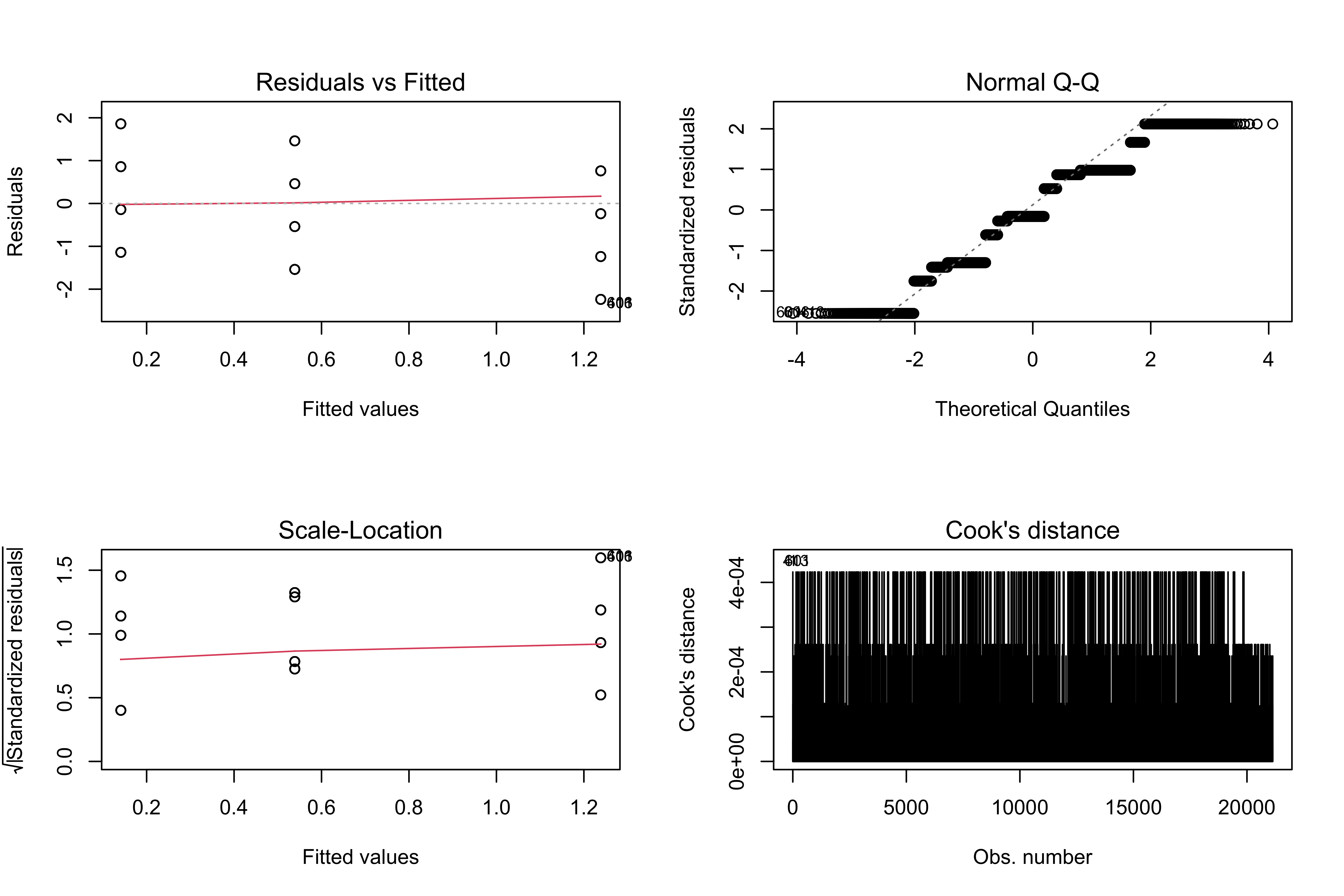


This new model, however, losses the datapoints from author-opposed reviewers, which seemed highly influential. However, this new regression produced a similar contrast between author-suggested reviewer and editor-suggested reviewer (contrast = 0.398, *t*(21,132) = 24.7, *p* < 0.001, Cohen’s *d* = 0.453). In fact, compared to the main text, the contrast now is larger and the effect size, given by Cohen's score is larger too (from main text: contrast = 0.364, *t*(23,954) = 27.4, *p* < 0.001, Cohen’s *d* = 0.415).

**References**

Gwet, K. L. (2008). “Computing inter-rater reliability and its variance in the presence of high agreement." *British Journal of Mathematical and Statistical Psychology*, 61, 29-48.
